# Identification of Quantitative Trait Loci for Leaf Rust and Stem Rust Seedling Resistance in Bread Wheat Using a Genome-Wide Association Study

**DOI:** 10.3390/plants11010074

**Published:** 2021-12-27

**Authors:** Alibek Zatybekov, Yuliya Genievskaya, Aralbek Rsaliyev, Akerke Maulenbay, Gulbahar Yskakova, Timur Savin, Yerlan Turuspekov, Saule Abugalieva

**Affiliations:** 1 Laboratory of Molecular Genetics, Institute of Plant Biology and Biotechnology, Almaty 050040, Kazakhstan; alexbek89@mail.ru (A.Z.); julia.genievskaya@gmail.com (Y.G.); yerlant@yahoo.com (Y.T.); 2 Faculty of Biology and Biotechnology, Al-Farabi Kazakh National University, Almaty 050038, Kazakhstan; 3 Laboratory of Phytosanitary Safety, Research Institute of Biological Safety Problems, Gvardeisky 080409, Kazakhstan; aralbek@mail.ru (A.R.); maulenbay.id@gmail.com (A.M.); y_gulbahar@mail.ru (G.Y.); 4 Department of Science, S. Seifullin Kazakh Agro Technical University, Nur-Sultan 010011, Kazakhstan; t.savin@kazatu.kz

**Keywords:** bread wheat, leaf rust, stem rust, resistance, association mapping

## Abstract

In recent years, leaf rust (LR) and stem rust (SR) have become a serious threat to bread wheat production in Kazakhstan. Most local cultivars are susceptible to these rusts, which has affected their yield and quality. The development of new cultivars with high productivity and LR and SR disease resistance, including using marker-assisted selection, is becoming an important priority in local breeding projects. Therefore, the search for key genetic factors controlling resistance in all plant stages, including the seedling stage, is of great significance. In this work, we applied a genome-wide association study (GWAS) approach using 212 local bread wheat accessions that were phenotyped for resistance to specific races of *Puccinia triticina* Eriks. (*Pt*) and *Puccinia graminis* f. sp. *tritici* (*Pgt*) at the seedling stages. The collection was genotyped using a 20 K Illumina iSelect SNP assay, and 11,150 polymorphic SNP markers were selected for the association mapping. Using a mixed linear model, we identified 11 quantitative trait loci (QTLs) for five out of six specific races of *Pt* and *Pgt*. The comparison of the results from this GWAS with those from previously published work showed that nine out of eleven QTLs for LR and SR resistance had been previously reported in a GWAS study at the adult plant stages of wheat growth. Therefore, it was assumed that these nine common identified QTLs were effective for all-stage resistance to LR and SR, and the two other QTLs appear to be novel QTLs. In addition, five out of these nine QTLs that had been identified earlier were found to be associated with yield components, suggesting that they may directly influence the field performance of bread wheat. The identified QTLs, including novel QTLs found in this study, may play an essential role in the breeding process for improving wheat resistance to LR and SR.

## 1. Introduction

Bread wheat, or common wheat (*Triticum aestivum* L.), is one of the main cereal crops cultivated around the world and is, thus, important for food security. Kazakhstan is among the ten largest exporters of wheat, with a volume of 11.4 thousand tons produced in 2019 [1]. According to the Bureau of National Statistics of Kazakhstan, the sown area under wheat was 12.2 million hectares in 2021, which represents about 76.7% of the total area used for cereal crops in the country [2]. One of the largest problems in wheat production all over the world is foliar diseases. Among fungal pathogens of wheat worldwide, *Puccinia graminis* f. sp. *tritici* (*Pgt*), causing stem rust (SR), and *Puccinia triticina* Eriks. (*Pt*), causing leaf rust (LR), are the most common [3,4,5]. LR and SR may cause more than 50% grain yield losses in susceptible wheat cultivars [6], while some aggressive strains, such as *Pgt* race Ug99, cause severe wheat yield losses of up to 90% [5,7].

*Pt* is an obligate biotrophic fungus that mainly infects the leaves at various growth stages, but it can also infect the leaf sheath and glumes [8]. *Pt* is a significant hindrance for wheat production, generally causing yield losses from 1% to 20% over a large area. However, if severe disease occurs prior to heading time, up to 90% of the wheat crop may be destroyed [9]. The occurrence of LR in Kazakhstan has been observed ever since wheat started to be cultivated on a wider scale in the early 1900s. The cultivation of susceptible cultivars resulted in epidemics of LR in 1 year out of 4 on average, affecting up to 5 million ha with yield losses of up to 25–30% [10,11]. *Pgt* causing SR is another important rust disease that is often considered as the most devastating of the wheat rust diseases, because it may cause complete crop loss over a large area within a short period of time [7]. In 2015 and 2016, a major SR epidemic occurred in the northern regions of Kazakhstan, as well as in the adjacent Omsk region of Russia, affecting approximately two million ha of wheat [11,12]. An SR epidemic occurred again in 2017–2018 in the northern and eastern regions of Kazakhstan, and it resulted not only in severe yield reduction, but also in lower grain quality [11,12,13,14]. During 2015–2018, disease severity and incidence in the main wheat-growing regions of Kazakhstan were up to 90% and 70%, respectively, and were substantially higher than in previous years [11,15].

One of the most effective ways to prevent wheat rust epidemics is the development of cultivars with durable resistance to pathogens. LR and SR resistance is controlled by a diverse group of genes, designated as *Lr* and *Sr*, respectively [16]. Nowadays, approximately 80 leaf rust resistance genes (*Lr*) and about 60 stem rust resistance genes (*Sr*) have been identified and described in bread wheat, durum wheat, and diploid wheat species [16]. Wheat rust resistance genes belong to one of two classes: seedling resistance genes (R) or the adult plant resistance (APR) genes, which are active only at the adult plant stage [16,17]. APR genes are considered potentially more durable, while R genes have a lack of durability [16,18]. R genes often encode nucleotide-binding site proteins and recognize specific pathogen effectors [16]. Due to their selective specificity to effectors, R genes are usually called race-specific genes. This specificity to particular races results in the greater effectiveness of R genes. R genes mostly have stronger effects but, on the other hand, they lose their strength after several years in the field [16]. There is also frequent emergence of new virulent pathogen races that overcome even the strongest resistance genes. For example, *Pgt* race Ug99 (also known as TTKSK) is insensitive to the *Sr31* resistance gene, which is highly effective against almost all *Pgt* races [7]. This means that the emergence of new virulent pathogen races restricts the durability and effectiveness of R and APR genes. It has been shown that the pathogen population structure on a particular territory is not constant and may change from year to year [15,19]. Therefore, there is a constant need for new sources of resistance in the wheat genome, including both R and APR genes.

Pathogen race composition is distinct in each region of the world and changes depending on many factors, including climatic conditions and human factors. Numerous studies on the race composition of *Pt* and *Pgt* in different countries [13,14,15,20,21,22] have been conducted to search for resistant germplasms [23,24,25] and QTLs [26,27,28].

In Kazakhstan, 38 *Pgt* races were identified and described in major wheat-growing areas in the country in 2015–2018 [15]. Among them, the most virulent races RFRTF and TKRTF were observed in the Kostanay and Akmola regions and, at the same time, were registered in the neighboring Omsk region in the Russian Federation [14]. The stem rust resistance genes *Sr11*, *Sr13*, *Sr22*, *Sr26*, *Sr31*, *Sr33*, and *Sr35* were confirmed to be effective against all *Pgt* races found in Kazakhstan. Although Ug99 has not been observed in Kazakhstan yet [15], recent reports have indicated the spreading of this race in the Middle East and a potential scenario of migration to Central and South Asia [5,29]. In Kazakhstan, 25 races of *Pt* were identified based on the assessments in all major wheat-growing regions of the country in 2018. The TQTMQ, TQKHT, and TRTHT races were the most common and were found in all studied populations [30].

Previously, QTLs for wheat seedling resistance to three common *Pgt* (TKRTF, PKCTC, and RKRTF) and three common *Pt* races (TQKHT, TRTHT, and TQTMQ) in Kazakhstan were identified using bi-parental mapping population Pamyati Aziaeva × Paragon [31]. Some of those QTLs were associated with known *Lr* and *Sr* genes, but several QTLs were novel, with high breeding potential. However, the linkage mapping (LM) used in that study has certain limitations that were attributed to a restricted level of genetic diversity defined by a pedigree of parental lines [32]. In comparison with linkage mapping, a genome-wide association study (GWAS) approach uses large germplasm collections with high genetic diversity. The wheat collection in this study was previously used for GWAS analysis of APR to leaf and stem rusts in southern Kazakhstan [33]. Therefore, an interesting question is - whether GWAS would facilitate the identification of unique race-specific QTLs not identified by LM? Additionally, the other aim of the study is to search for commonality/differences in APR and R genes using GWAS for the same studied spring wheat collection. The identification of race-specific QTLs would be beneficial for future pyramiding of rust resistance genes in order to extend the effectiveness of wheat production and prevent rust epidemics in the territory of Kazakhstan.

## 2. Results

### 2.1. Screening of Infection Type of Pt and Pgt Races

**Figure 1:**
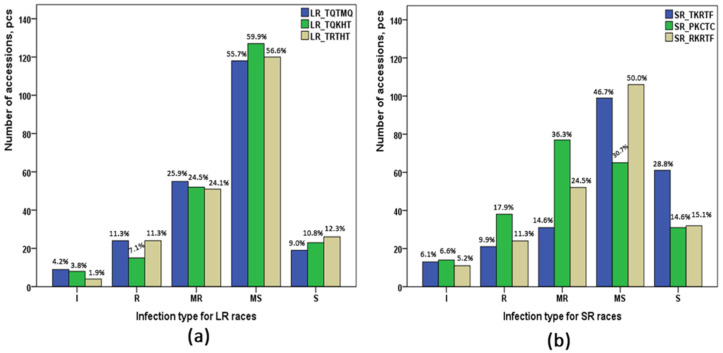
Summary of the reactions of 212 wheat cultivars and breeding lines to races of *Puccinia triticina* (**a**) and *Puccinia graminis* f. sp. *tritici* (**b**). I: immune; R: resistant; MR: moderately resistant; MS: moderately susceptible; S: susceptible.

In general, the genotypes of the studied collection were moderately susceptible to three *Pt* races (Figure 1): 55.7% for the race TQTMQ, and 56.6% and 59.9% for races TRTHT and TQKHT, respectively. Susceptible infection type was observed in 8.9%, 10.9%, and 12.3% of the collection. The cultivars Saratovskaya 29 (Russia) and Lutescens 1082 (Kazakhstan) were susceptible to all three races of *Pt* that were tested. Resistant infection type was observed for 15 (7.1%) accessions to the race TQKHT and for 24 (11.3%) accessions to the races TQTMQ and TRTHT. Cultivar Lutescens 1193 (Russia) demonstrated complete resistance to all three races of *Pt*.

Evaluation of resistance to three *Pgt* races resulted in responses similar to those observed for *Pt* races. The majority of the collection was moderately susceptible for races TKRTF (46.7%) and RKRTF (50%) (Figure 1). As for the race PKCTC, most of the genotypes were of a moderately resistant infection type (36.3%, 77 accessions). Six cultivars revealed immunity to all three races of *Pgt*. These are accessions IR-38, IR-53, and E-795 of local breeds; Agent and Gatcher from America; and Seri 82 from Australia. Screening of seedling resistance identified three local cultivars (Karabalykskaya 25, Karabalykskaya 92, and Oskemen), which had a susceptible infection type to all three races of *Pgt*.

**Figure 2:**
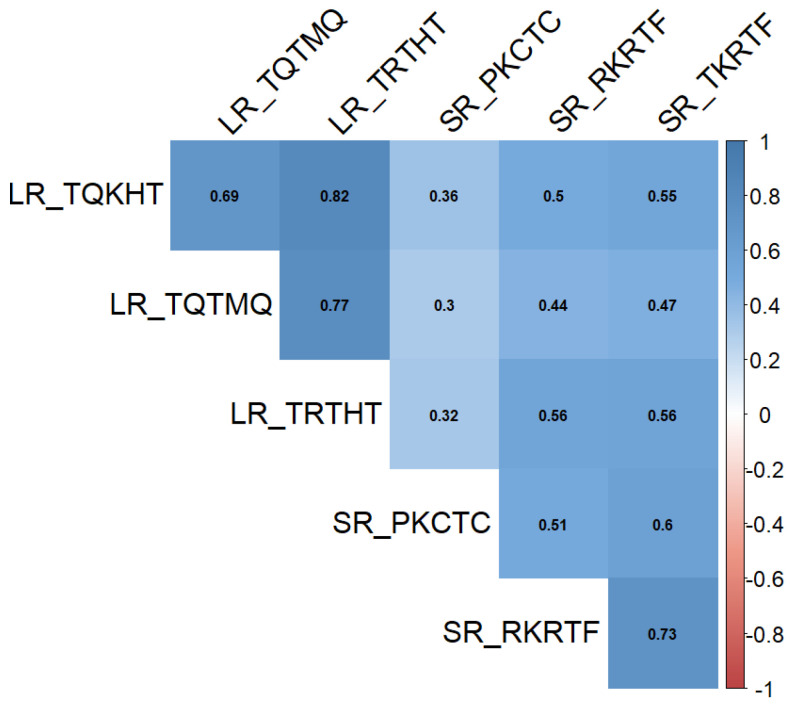
Pairwise correlation analysis of leaf rust (LR) and stem rust (SR) infection type.

Pearson correlation analysis revealed a strong positive correlation (*p* < 0.001) of infection type among all *Pt* and *Pgt* races (Figure 2).

Two-way ANOVA revealed a strong significant effect (*p* < 0.001) of two factors (genotype and race), both separately and combined, on the resistance to LR and SR (Table 1). The broad-sense heritability (*H*^2^) of resistance to LR was higher than for resistance to SR (Table 1).

The correlations of LR and SR resistances between seedling and adult stages [33] were highly significant (Table 2). The average correlation index value for *Pt* races was higher for APR_LR (0.642) than for APR_SR (0.458). Similarly, the average correlation index value for *Pgt* races was higher for APR_SR (0.634) than for APR_LR (0.468).

### 2.2. Genotyping Results and Analysis of the Population Structure

Genotypic data of 212 common wheat accessions were compiled for 11,510 polymorphic SNP markers that were selected for the GWAS. The distribution of SNP markers among genomes was 2186 for A, 2955 for B, and 414 for D. The remaining 5955 markers in the 20 K array had unknown genomic positions. Chromosome 2B had the largest number of markers (640 SNP), and chromosome 7A was the longest chromosome (216.0 cM). The average SNP density for the three genomes was 1.6 markers/cM. The highest density was observed for genome B, with an average distance of 0.3 cM between neighboring markers. Generally, the density of the D genome was about seven times less than those of genomes A and B [33]. In average, linkage disequilibrium (LD) decayed at 14.9 cM for the whole genome at R2 of 0.1. For the subgenomes, the LD decay at 7.1 and 5.3 cM in the A and B genomes, respectively; for the D genome, the LD extends to 19.2 cM [33]. Based on the results of STRUCTURE and STRUCTURE Harvester analyses, the Q matrix was developed for three subpopulations, as suggested in Genievskaya et al. [33]. The generated Q matrix was used as a covariate matrix for MLM + Q + K in TASSEL.

### 2.3. Association Mapping

In total, 11 marker-trait associations with significant *p*-values were identified for the race-specific seedling resistance to LR and SR. The identified marker-trait associations were located on eight chromosomes (1A, 1B, 1D, 2A, 4B, 5B, 6A, and 7A). Manhattan and QQ plots for all races are provided in Table S1. Physical positions, effects, and R2 values for identified SNPs associated with race-specific seedling resistances to LR and SR were given in Table 3. All marker-trait associations were designated as QTLs and positioned on the genetic map along with approximate positions of potential candidate genes for LR and SR resistance (Table 3 and Table S2; Figure S1). Among the identified QTLs, three had associations with resistance to only LR. The other eight identified QTLs were associated with resistance for both diseases (LR and SR).

All eleven QTLs responsible for the resistance to LR were associated with the race TRTHT. One of the QTLs was associated with the combination of races TQKHT and TRTHT, and six QTLs with a combination of all three races (Table 3). Eight out of eleven LR QTLs were associated with *Pgt* race TKRTF, and two with the combination of races TKRTF and RKRTF (Table 3). None of the QTLs were associated with PKCTC (Table 3). Notably, there were no genetic factors common to this work and that of a report that was based on the study of the same *Pt* and *Pgt* races using bi-parental mapping population Pamyati Azieva × Paragon [31].

The QTLs identified in this study were analyzed and compared with the QTLs previously reported for APR resistance to LR and SR that were identified using GWAS data from RIBSP 2018–2019 [33] and the QTLs previously reported to be associated with yield-related traits identified using GWAS data from northern Kazakhstan 2018–2020 [34] (Table 4). In addition, the location of each identified QTL was compared to the genetic positions of known *Lr* and *Sr* genes (Table 4). In total, three candidate *Lr* genes and 9 QTLs were found for 11 QTLs associated with LR resistance in this study. In the analysis of QTLs for SR resistance, there were no similarities among the genetic locations of known *Sr* genes and one similarity with previously identified QTLs (Table 4).

## 3. Discussion

The results indicate a lack of QTLs identified both at the seedling stage in this GWAS study and a previous LM study using Pamyati Azieva × Paragon RILs [31], suggesting different responses between two types of genetic materials with the same *Pt* and *Pgt* races. Notably, both the APR for LR and SR in LM and GWAS populations were tested in the same environment and for the same years (RIBSP, 2018–2019). It can be hypothesized that the difference was probably determined by the specificity of the LM population’s reaction due to the pedigree restricted by two parental pools. Additionally, unlike in the LM study [31] where no correlation was registered between race-specific seedling resistance and APR, the correlation of resistance between two growth stages was found to be highly significant (*p* < 0.0001) in this study.

By contrast, it was found that 9 out of 11 QTLs identified in this study had been previously identified in an APR GWAS using data from RIBSP in 2018–2019 [33] (Table 4), suggesting that these are QTLs for all-stage resistance. Notably, all nine QTLs in this comparative assessment were characterized by similar directions of QTL effects, i.e., toward either resistance or susceptibility. In addition, five out of these nine QTLs were earlier found to be associated with yield components [34] (Table 4), suggesting that these QTLs might directly influence the field performance of bread wheat. The comparative evaluation of all eleven identified QTLs suggests that two QTLs (*QLr.ipbb-1A.3* and *QLr.ipbb-1B.5*) were presumably novel, as they were not reported in previously published LR and SR studies (Table 4).

The majority of the studied collection had shown moderately susceptible IT at the seedling stage to all races of *Pt* and *Pgt*, except for the *Pgt* race PKCTC, where most of the accessions were moderately resistant (Figure 1). The ANOVA showed a more significant influence of wheat genotype as compared to race type on the resistance to both diseases (Table 1), suggesting the significant involvement of genetic factors in the resistance to all six studied races.

### 3.1. Patterns of Identified QTLs for Leaf and Stem Rust Resistances

Multiple occurrences of most QTLs were associated with LR resistance in different growth stages and environments. In particular, six identified QTLs appeared to be efficient in LR resistance to all three specific races, which is an indication of the broad stability of these loci. Three of those six QTLs (*QLr.ipbb-2A.2*, *QLr.ipbb-4B.2*, and *QLr.ipbb-6A.2*) showed effects toward LR resistance, while the effects in the remaining three QTLs (*QLr.ipbb-1B.2*, *QLr.ipbb-6A.6,* and *QLr.ipbb-7A.2*) were toward susceptibility (Table 3). The latter three QTLs can be selected for the rapid elimination of susceptible cultivars via breeding efforts. Interestingly, the *QLr.ipbb-4B.2* was positioned in the vicinity of genes *Lr12* (70.9 cM) [35], *Lr31* (70.9 cM) [36], and *Lr49* (81.5 cM) [37] on chromosome 4B (Table 5). This result confirms the previously reported effects of *Lr12* [35] in northern and southeastern Kazakhstan [11]. *QLr.ipbb-6A.2* was closely mapped to the SNP marker S16_50275005 of LR identified by Juliana et al. [38], which is adjacent to the *Traes_6AS_EB7270F83* gene with a predicted LRR (leucine-rich repeat) receptor-like STPK (serine/threonine-protein kinase) function. The QTLs *QLr.ipbb-1B.5*, *QLr.ipbb-2A.2*, and *QLr.ipbb-4B.2* are located at the same position as *QTLs 1B_1, 2A_2*, and *4B_3* identified in the GWAS for LR by Gao et al. [39]. Additionally, *QLr.ipbb-7A.2* was in the vicinity of *QTL 7A_3* (~2 cM) [39]. The QTL QLr.ipbb-2A.2 was positioned approximately 1.4 cM away from SNP IWA574, which was previously found to be associated with seedling resistance to *Pt* race TBDJ [40]. The *QLr.ipbb-5B.1* was in a similar genetic position to *QLr.fcu-5BL* associated with LR field resistance [26].

None of the eight SR QTLs identified in this study (Table 4) were located in the vicinity of known *Sr* genes. Additionally, all eight SR QTLs at the seedling stage conferred seedling resistance to LR and adult resistance to SR, confirming that the identified QTLs are expressed in both the seedling and adult stages. The comparative evaluation of the identified SR QTLs in this study with known SR resistant factors also identified several examples of pleiotropic effects. For instance, the location of *QSr.ipbb-1B.2* (race TKRTF) was adjacent to the genetic position of IWB42604 that was associated with seedling resistance to the TRTTF race [43]. Two QTLs responsible for the resistance to SR were previously identified by Genievskaya et al. [33] at the adult plant stage (Table 4). A survey of the associated literature comprising many studies on bread and durum wheat and their resistance to LR and SR suggests that pleiotropy is a common scenario [44,45]. Hence, the finding in this study may positively impact the development of high-yielding wheat germplasm with the resistance to *Pt* and *Pgt* races via the application of marker-assisted selection.

### 3.2. Comparison of the Physical Positions of SNPs in Quantitative Trait Loci and Protein-Coding Genes

Among eleven marker-trait associations identified in this work (Table 4), three protein-coding genes are potentially directly involved in determining the resistance to rust pathogens. The position of one of those genes, *TraesCS4B02G328500*, coding for the major facilitator superfamily (MFS) domain-containing protein, overlapped with the positions of *QLr.ipbb-4B.2* and *QSr.ipbb-4B.1*. The MFS transporter has previously been reported to participate in the secretion of fungi toxin, which affects host species [46]. The position of the *TraesCS7A02G250500* gene, coding for L-ascorbate peroxidase 6, overlaps with those of *QLr.ipbb-7A.2* and *QSr.ipbb-7A.2*. Gou and co-authors (2015) proposed that the phosphorylation of ascorbate peroxidase by the *Wheat Kinase START 1* (*WKS1.1*) gene reduces the ability of the cells to detoxify reactive oxygen species, thus contributing to promoting cell death [47]. This response takes several days longer than typical hypersensitive cell death responses, thus allowing the limited pathogen growth and restricted sporulation that is characteristic of the *WKS1* partial resistance response to *Puccinia striiformis* [47]. The position of the other gene, *TraesCS7A02G389100*, coding for the Rab-GAP TBC domain-containing protein, physically overlapped with the genetic positions of *QLr.ipbb-7A.2* and *QSr.ipbb-7A.3*. This protein has positive or negative effects on the immune response of wheat to infection by rust pathogens depending on its levels [48]. Although the functions of the protein-coding genes associated with SNPs, as mentioned above, do not directly explain the genetic mechanism of resistance to the studied rusts, they still indicate their potential involvement in the complex processes of plant resistance.

## 4. Materials and Methods

### 4.1. Genetic Material

A total of 212 (Table S3) wheat cultivars and breeding lines were selected and evaluated for their response upon exposure to *Pt* and *Pgt* races at the seedling stage. The collection included 88 commercial and prospective breeding cultivars from Kazakhstan and Russia, including 64 cultivars approved by the State Seed Trials Commission for use in the territory of Kazakhstan; 38 cultivars from Europe provided by the John Innes Centre, United Kingdom; and 86 cultivars and lines from Kazakhstan, Russia, USA, Canada, Mexico, Germany, and Australia provided by the Research Institute of Biological Safety Problems (RIBSP, Gvardeisky, South Kazakhstan) [33]. Most of the cultivars and lines from Kazakhstan and Russia originated from locally made crosses, though a few originated from the Kazakhstan–Siberia Network for Spring Wheat Improvement shuttle breeding program.

### 4.2. Seedling LR and SR Evaluation

The evaluation of race-specific resistance was conducted at the seedling stage under greenhouse conditions at the RIBSP in 2019. For the resistance assessment, seedlings of the studied collection were separately inoculated with three races of *P. triticina* (TQTMQ, TQKHT, and TRTHT) and three races of *P. graminis* (TKRTF, PKCTC, and RKRTF) with different levels of virulence to *Lr* and *Sr* genes, respectively [15,30]. Seeds of each accession were sown in plastic pots (6 seeds per pot) in two replicates for each rust race. Before the inoculation, urediniospores of pathogen races (stored in a refrigerator at −80 °C) were heated at 40 °C for 10 min, followed by watering in a humid chamber at 20 °C for 2 h, containing a 23.5% KOH solution (80% relative humidity) [49]. Urediniospores were then suspended in light mineral oil (Soltrol 170), and each pot with wheat seedlings was individually inoculated by spraying with races of *Pt* and *Pgt* onto the fully expanded primary leaves of 7–9-day-old seedlings. Seedlings were incubated in a humid chamber in the dark at 18 ± 2 °C and 100% humidity for 14 h and then exposed to fluorescent light for 3–4 h. The inoculated plants were placed in greenhouse boxes, with favorable conditions (22 ± 2 °C for stem rust, 18 ± 2 °C for leaf rust) and illumination (10–15 thousand lux, light period 16 h) [26,50,51]. The resistance of the studied collection was assessed two weeks after inoculation according to the Stakman et al. infection type scale [52]. The infection type values for each combination of wheat accession and pathogen race was determined as an average for 6 plants in the pot. In order to use the Stakman et al. scale in the GWAS, the 0–4 scale was converted into a 0–9 linear scale as proposed by Zhang et al. [53]. The average resistant values for two replications were further used in GWAS.

The statistical analysis included correlation analysis and analysis of variance (two-way ANOVA) using the SPSS 22.0 (https://www.ibm.com/support/pages/spss-statistics-220-available-download, accessed on 13 July 2021) and STATISTICA 10.0 (http://statsoft.ru/resources/support/download.php, accessed 21 July 2021) software packages. Variance components (%) were determined by the division of phenotypic variance due to each component on the total phenotypic variance. The broad-sense heritability (*H*^2^), describing the proportion of phenotypic variation due to genetic factors, was calculated by the following formula:

$$H^{2}=\frac{\sigma_{g}^{2}}{\sigma_{p}^{2}}$$

where $\sigma_{g}^{2}$ is phenotypic variance explained by the genotype and $\sigma_{p}^{2}$ is the total phenotypic variance (sum of genotype variance, race variance, genotype × race variance, and residuals variance) [41].

### 4.3. DNA Extracting and Genotyping

Total DNA was isolated from the seedlings of wheat accessions according to Dellaporta et al. [42]. The DNA concentration for each sample was adjusted to 50 ng/mL. The panels of the studied collection were genotyped using 20K Illumina iSelect SNP assay at the TraitGenetics GmbH (Gatersleben, Germany). SNP genotyping was performed using the Illumina Genome Studio software version V2011.1 (Illumina Inc., San Diego, CA, USA, 2018). A total of 11,510 SNP markers [33] were selected after removing all monomorphic markers and markers with a minor allele frequency (MAF) <0.05. Accessions with more than 10% missing data were also removed.

### 4.4. Association Mapping

The analysis of the population structure was carried out using STRUCTURE (v2.3.4.) software with a Bayesian Markov chain Monte Carlo (MCMC) approach based on the admixture and correlated frequency models [54]. The numbers of hypothetical groups ranging from K = 1 to K = 10 were assessed using 50,000 burn-in iterations, followed by 100,000 recorded iterations. The output from STRUCTURE was analyzed for the delta *K* value (Δ*K*) in STRUCTURE HARVESTER [55].

Using *K* = 5 values, the Q-matrix for the five identified clusters was developed. GWAS was conducted using TASSEL 5.0 (v20191212) [56] based on the mixed linear model (MLM) with the kinship (K) and Q matrices (MLM + K + Q) [57]. For confirmation of the correction due to K and Q matrices, the distribution lines in each quantile–quantile plot were analyzed. Significant marker-trait associations were selected after the application of a threshold at *p* < 0.0001. The positions and sequences of SNP markers were obtained from the 90K Array Consensus map of the common wheat genome [58]. For markers with unknown positions in the 90K Array Consensus map, the CSS POPSEQ 2014 map [59], available at the Triticeae Toolbox (2020), was used. For several significant marker-trait associations linked to each other, the SNP with the lowest *p*-value was chosen. For the search of protein-coding genes that overlap with identified significant marker–trait associations, the sequence of each marker was inserted into the BLAST tool [60] of Ensembl Plants [61] and compared with the reference genome of *T. aestivum*. The genetic map was constructed using MapChart v.2.3 software [62].

## 5. Conclusions

A GWAS of 212 bread wheat accessions inoculated with three races of *P. triticina* (TQTMQ, TQKHT, and TRTHT) and three races of *P. graminis* (TKRTF, PKCTC, and RKRTF) at the seedling stage of growth, resulted in the identification of eleven marker-trait associations for LR and SR resistance. Nine out of the eleven identified QTLs were previously reported in a GWAS using the same collection with assessment at the adult plant stage in a natural infection field of southern Kazakhstan in 2018–2019. Correspondingly, it was concluded that these nine identified QTLs were effective for all-stage resistance to LR and SR, and the two other QTLs appear to be novel and were effective at the seedling growth stage for the LR resistance. Five out of these nine QTLs were earlier found to be associated with yield components, suggesting that these QTLs might directly influence the field performance of bread wheat. In addition, the alignment of SNPs in QTLs to the sequencing data of a hexaploid wheat physical map using the Ensemble platform suggests the direct involvement of at least three protein-coding genes in determining the resistance to rust pathogens. The obtained results can be further validated and potentially used in marker-assisted selection for the construction of new highly productive cultivars resistant to LR and SR.

## Figures and Tables

**Table 1:** ANOVA analyses of infection type of LR and SR.

Disease	Factor	df	SS	MS	F-Value	*p*-Value	Genotype (*H*^2^) %	Race %	Genotype × Race %
LR	Genotype	211	5222	24.8	20.9	<2 × 10^−16^	74.60	0.47	14.19
	Race	2	33	16.7	14.1	9.97 × 10^−07^			
	Genotype × Race	422	993	2.4	2.0	1.99 × 10^−15^			
	Residuals	636	752	1.2					
SR	Genotype	211	5852	27.7	16.9	<2 × 10^−16^	63.35	3.18	22.14
	Race	2	294	146.9	89.4	<2 × 10^−16^			
	Genotype × Race	422	2045	4.9	3.0	<2 × 10^−16^			
	Residuals	636	1046	1.6					

Notes: LR, leaf rust; SR, stem rust; df, degree of freedom; SS, a sum of squares; MS, mean squares; *H*^2^, the broad-sense heritability.

**Table 2:** Correlation among race-specific seedling resistance and adult plant resistance (APR) to leaf rust (LR) and stem rust (SR) in the studied collection.

	APR LR	APR SR	LR_TQTMQ	LR_TQKHT	LR_TRTHT	SR_TKRTF	SR_PKCTC
**APR SR**	0.5580 ***						
**LR_TQTMQ**	0.6037 ***	0.4290 ***					
**LR_TQKHT**	0.6549 ***	0.4748 ***	0.6936 ***				
**LR_TRTHT**	0.6672 ***	0.4994 ***	0.7734 ***	0.8155 ***			
**SR_TKRTF**	0.5563 ***	0.6838 ***	0.4687 ***	0.5494 ***	0.5633 ***		
**SR_PKCTC**	0.2702 ***	0.5607 ***	0.3002 ***	0.3590 ***	0.3228 ***	0.5968 ***	
**SR_RKRTF**	0.5483 ***	0.6563 ***	0.4415 ***	0.5033 ***	0.5566 ***	0.7261 ***	0.5115 ***

Notes: *** *p* < 0.001; APR LR and APR SR are the average value of field data of 2018–2019 years.

**Table 3:** List of quantitative trait loci (QTLs) for race-specific seedling resistance to leaf rust (LR) and stem rust (SR).

#	SNP Marker	Chr.	Pos^1^. (cM)	Pos^2^. (bp)	Leaf Rust (LR)			Stem Rust (SR)			Allele	Effect	R2
					TQTMQ	TQKHT	TRTHT	TKRTF	PKCTC	RKRTF			
1	Kukri_c41943_535	1A	38.1	13,230,954	-	-	1.29 × 10^−05^	-	-	-	A	1.61	0.10
2	TA001473-0980	1B	62.4	54,075,692	-	1.32 × 10^−05^	1.37 × 10^−05^	-	-	-	A	2.41	0.09
3	BS00078431_51	1B	70.8	346,871,402	3.61 × 10^−05^	9.85 × 10^−06^	5.45 × 10^−08^	7.21 × 10^−07^	-	-	C	1.72	0.11
4	BS00063511_51	1D	167.1	485,708,706	-	-	2.46 × 10^−05^	5.33 × 10^−05^	-	-	A	1.46	0.08
5	BobWhite_c14476_80	2A	102	86,901,274	2.09 × 10^−06^	3.23 × 10^−06^	6.33 × 10^−08^	2.47 × 10^−07^	-	8.40 × 10^−05^	A	−1.67	0.11
6	Excalibur_c27349_166	4B	77.9	619,448,536	9.81 × 10^−08^	1.07 × 10^−05^	2.43 × 10^−11^	1.59 × 10^−05^	-	-	C	−1.93	0.15
7	GENE-2307_1216	5B	147.4	531,888,962	-	-	4.50 × 10^−05^	-	-	-	G	1.53	0.08
8	wsnp_Ex_rep_c68175_66950387	6A	31.9	13,600,765	1.67 × 10^−05^	4.37 × 10^−05^	8.37 × 10^−07^	1.87 × 10^−05^	-	-	C	1.70	0.09
9	TA003021-1057	6A	56.1	34,974,650	9.58 × 10^−06^	3.51 × 10^−05^	1.25 × 10^−07^	6.51 × 10^−06^	-	-	A	−1.83	0.10
10	BobWhite_c24063_231	7A	127.7	232,746,015	1.46 × 10^−06^	4.24 × 10^−05^	3.42 × 10^−07^	4.83 × 10^−07^	-	7.16 × 10^−05^	C	1.82	0.10
11	TA003458-0086	7A	133.9	565,347,833	-	-	5.44 × 10^−06^	3.39 × 10^−06^	-	-	C	1.77	0.10

Notes: Chr., chromosome; Pos^1^., positions according to 90K Array Consensus map; Pos^2^., positions according to RefSeq v1.0; R2, part of the phenotype affected by the QTL.

**Table 4:** Comparison of quantitative trait loci (QTLs) of seedling resistance to leaf rust (LR) and stem rust (SR) identified in this study with previously described QTLs and candidate *Lr* and *Sr* genes.

#	SNP Marker	QTL ref	Candidate Gene(s)	Overlapping Gene(s)	Protein	Orthologue Gene(s)	Identity (%)
1	Kukri_c41943_535	*QLr.ipbb-1A.3*	-	*TraesCS1A02G027800*	Uncharacterized protein	TRIUR3_10793 (*T. urartu*)	97.8
2	TA001473-0980	*QLr.ipbb-1B.5*	-	*TraesCS1A02G071800*	Uncharacterized protein	AET1Gv20182900 (*A. tauschii*)	99.0
3	BS00078431_51	*QLr.ipbb-1B.2* *; *QSr.ipbb-1B.2* *; *QTKW.ta.ipbb-1D* †, *QNPS.ta.ipbb-1B* †	-	*-*	-	-	-
4	BS00063511_51	*QLr.ipbb-1D.2* *; *QSr.ipbb-1D.2*; *QTKW.ta.ipbb-1D* †, *QNPS.ta.ipbb-1B* †	-	*TraesCS1D02G439800*	Trimethylguanosine synthase	AET1Gv21018700 (*A. tauschii*)	96.3
5	BobWhite_c14476_80	*QLr.ipbb-2A.2* *; *QSr.ipbb-2A.3*	-	*TraesCS2A02G141400*	Cation efflux protein	TRITD_2Av1G039940 (*T. turgidum*)	99.0
6	Excalibur_c27349_166	*QLr.ipbb-4B.2* *; *QSr.ipbb-4B.1; QTKW.ta.ipbb-4B* †	*Lr12* [35], *Lr31* [36], *Lr49* [37]	*TraesCS4B02G328500*	MFS domain-containing protein	TRITD_4Bv1G186380 (*T. turgidum*)	99.7
7	GENE-2307_1216	*QLr.ipbb-5B.1* *	-	*TraesCS5D02G505900*	Uncharacterized protein	AET5Gv21130000 (*A. tauschii*)	100
8	wsnp_Ex_rep_c68175_66950387	*QLr.ipbb-6A.6* *; *QSr.ipbb-6A.3*	-	*TraesCS6D02G032300*	Protein kinase domain-containing protein	AET6Gv20067300 (*A. tauschii*)	99.9
9	TA003021-1057	*QLr.ipbb-6A.2* *; *QSr.ipbb-6A.1* *; *QTKW.ta.ipbb-6A* †, *QNPS.ta.ipbb-6A* †	-	*-*	-	-	-
10	BobWhite_c24063_231	*QLr.ipbb-7A.2* *; *QSr.ipbb-7A.2*	-	*TraesCS7A02G250500*	L-ascorbate peroxidase 6	TRITD_7Av1G095170 (*T. turgidum*)	99.6
11	TA003458-0086	*QLr.ipbb-7A.2* *; *QSr.ipbb-7A.3*; *QNPS.ta.ipbb-7A* †, *QTKW.ta.ipbb-7A* †	-	*TraesCS7A02G389100*	Rab-GAP TBC domain-containing protein	TRITD7Av1G209600 (*T. turgidum*)	100.0

* Identified in Genievskaya et al. [33]. † Identified in Amalova et al. [34].

**Table 5:** Virulence/avirulence pattern of pathogen races used in the study based on the nomenclature by Long and Kolmer [41] for LR and by Roelfs and Martens [42] for SR.

Disease (Pathogen)	Race	Avirulent (Effective) Genes	Virulent (Ineffective) Genes
LR (*Puccinia triticina* Eriks.)	TQTMQ	*Lr24*, *26*, *20*, *25*, *14a*, *15*	*Lr1*, *2a*, *2c*, *3*, *9*, *16*, *3ka*, *11*, *17*, *30*, *19*, *29*, *2b*, *3bg*
	TQKHT	*Lr24*, *26*, *3ka*, *19*, *25*	*Lr1*, *2a*, *2c*, *3*, *9*, *16*, *11*, *17*, *30*, *20*, *29*, *2b*, *3bg*, *14a*, *15*
	TRTHT	*Lr24*, *19*, *25*	*Lr1*, *2a*, *2c*, *3*, *9*, *16*, *26*, *3ka*, *11*, *17*, *30*, *20*, *29*, *2b*, *3bg*, *14a*, *15*
SR (*Puccinia graminis* Pers. f. sp. *tritici* Eriks. & E. Henn.)	TKRTF	*Sr11*, *30*, *24*, *31*	*Sr5*, *21*, *9e*, *7b*, *6*, *8a*, *9g*, *36*, *9b*, *17*, *9a*, *9d*, *10*, *38*, *Tmp*, *McN*
	PKCTC	*Sr21*, *11*, *36*, *9b*, *30*, *24*, *31*, *38*	*Sr5*, *9e*, *7b*, *6*, *8a*, *9g*, *17*, *9a*, *9d*, *10*, *Tmp*, *McN*
	RKRTF	*Sr9e*, *11*, *30*, *24*, *31*	*Sr5*, *21*, *7b*, *6*, *8a*, *9g*, *36*, *9b*, *17*, *9a*, *9d*, *10*, *38*, *Tmp*, *McN*

## Data Availability

No data available.

## Supplementary Materials

The following are available online at https://www.mdpi.com/article/10.3390/plants11010074/s1; Figure S1: Genetic map of the location of the identified QTLs associated with seedling resistance to *Pt* and *Pgt* races. Table S1: Graphical representation of GWAS results using TASSEL software based on MLM+Q+K method, Table S2: Complete data of GWAS results for race-specific seedling resistance to leaf rust and stem rust, Table S3: List of common wheat cultivars and breeding lines used in the current study.
