# Supplementary figures and images for: Identification of Quantitative Trait Loci for Leaf Rust and Stem Rust Seedling Resistance in Bread Wheat Using a Genome-Wide Association Study
Source: Plants (Basel). 2021 Dec 27;11(1):74. doi: 10.3390/plants11010074 (PMC8747073; doi:10.3390/plants11010074)

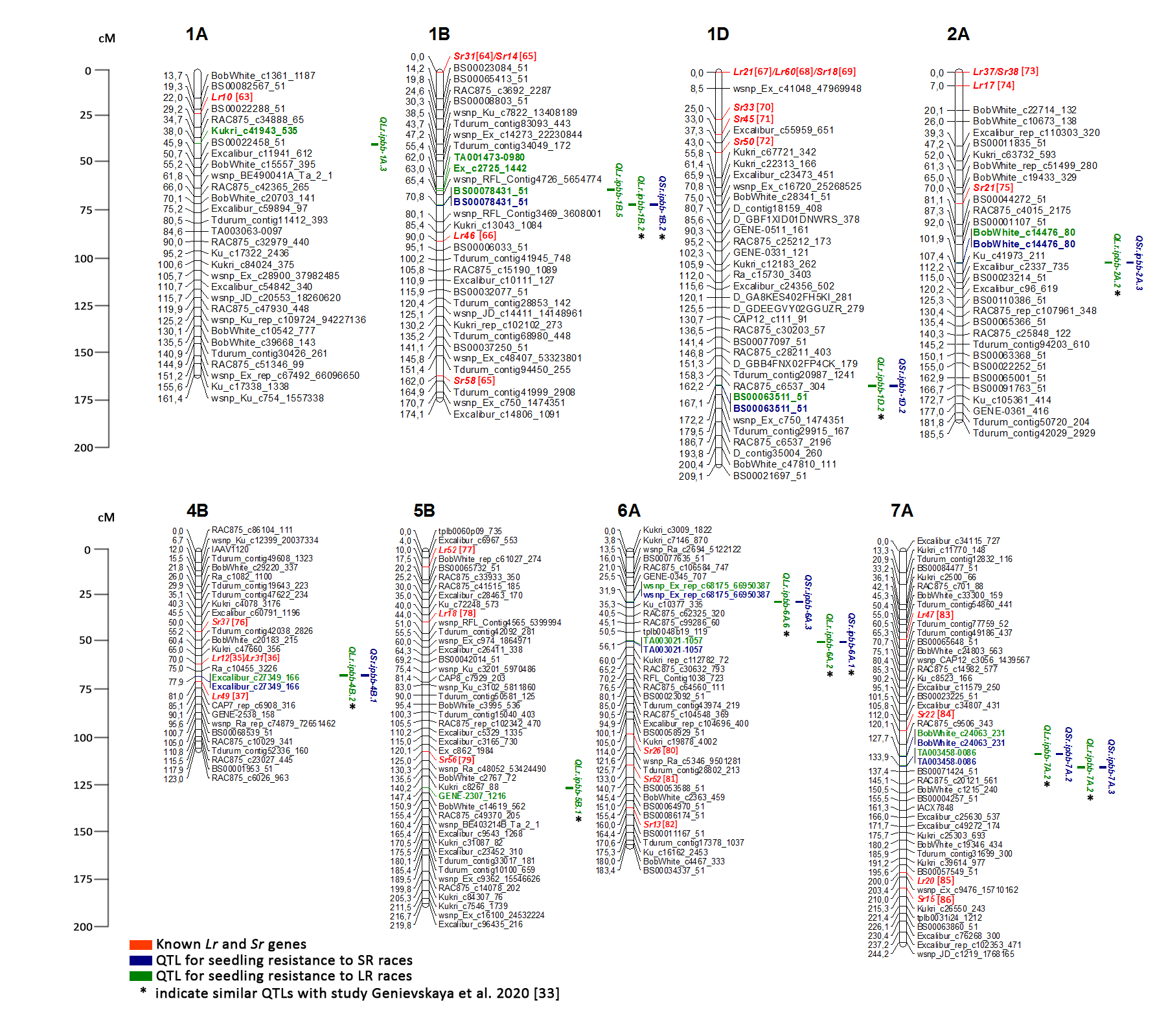

Supplement: Supplementary file 1 [file plants-11-00074-s001.zip › plants-1489474-Figure S1.docx.png]
